# One year overall survival of wilms tumor cases and its predictors, among children diagnosed at a teaching hospital in South Western Uganda: a retrospective cohort study

**DOI:** 10.1186/s12885-023-10601-2

**Published:** 2023-03-02

**Authors:** Eddymond Ekuk, Charles Newton Odongo, Leevan Tibaijuka, Felix Oyania, Walufu Ivan Egesa, Felix Bongomin, Raymond Atwiine, Moses Acan, Martin Situma

**Affiliations:** 1 Department of Surgery, Mbarara University of Science and Technology, Faculty of Medicine, Mbarara, Uganda; 2 Department of Anatomy, Faculty of Medicine Soroti University, Soroti, Uganda; 3 Department of Pediatrics, Faculty of Clinical Medicine and Dentistry, Kampala International, Kampala, Uganda; 4 Department of Microbiology, Mycology, and Immunology, Internal Medicine Gulu University, Gulu, Uganda

**Keywords:** Wilms tumor, Overall survival, Event-free survival, Predictors of survival, Histology characteristics

## Abstract

**Background:**

Wilms tumor (WT) is the second most common solid tumor in Africa with both low overall survival (OS) and event-free survival (EFS) rates. However, no known factors are predicting this poor overall survival.

**Objective:**

The study was to determine the one-year overall survival of WT cases and its predictors among children diagnosed in the pediatric oncology and surgical units of Mbarara regional referral hospital (MRRH), western Uganda.

**Methodology:**

Children’s treatment charts and files diagnosed and managed for WT were retrospectively followed up for the period between January 2017 to January 2021. Charts of children with histologically confirmed diagnoses were reviewed for demographics, clinical and histological characteristics, as well as treatment modalities.

**Results:**

One-year overall survival was found to be 59.3% (95% CI: 40.7–73.3), with tumor size greater than 15 cm (p 0.021) and unfavorable WT type (p 0.012) being the predominant predictors.

**Conclusion:**

Overall survival (OS) of WT at MRRH was found to be 59.3%, and predictive factors noted were unfavorable histology and tumor size greater than 115 cm.

## Introduction

Worldwide, WT affects about 8.1 per million children [1]. United States has the highest number of about 650 children newly diagnosed with WT annually [2]with a high overall survival (OS) of 85%-90% for localized disease, in children who underwent multimodal treatment of chemotherapy, surgery, and radiotherapy [3]. For children with stage one epithelial-predominant favorable histology OS is even excellent, at 100% [4]. The number of children diagnosed in Africa is equally high, with an incidence of 9.8 age-specific rates per million (ASR/million) [5], conversely with a poor OS of 25% [6]. In East Africa, Kenya has a relatively good survival rate of 67% [7] compared to Uganda at 43.6% [8].

In Nigeria, the stage of WT at diagnosis, age at diagnosis, histology type, frequent treatment interruptions, and size of the tumor were factors predicting OS of children diagnosed with WT [9]. In Germany, a WT size greater than 10 cm was associated with poor OS [10].

WT treatment involves multi-modal therapy with chemotherapy, radiation, and surgery [11]. However, surgery is the cornerstone of treatment [9], as it provides local primary tumor control, necessitates tumor staging, as well as controls metastatic spread and extension into the vessels [12]. The sequence of multimodal therapy depends on the treatment protocol employed. The Société Internationale D’oncologie Pediatrique (SIOP) protocol starts with neoadjuvant chemotherapy, followed by surgery, and then postoperative chemotherapy [13]. This protocol is advantageous because it debulks the tumor before nephrectomy, thereby minimizing risks of spillage intraoperatively. However, this protocol also carries a potential risk of a high burden of chemotherapy, in case chemotherapy is administered to renal tumors other than WT [14]. The Children’s Oncology Group (COG) recommends upfront surgery [15].

The target of this study was to assess predictors of one-year OS in children diagnosed with WT.

## Methods

### Study design

This was a retrospective cohort study of 41 children diagnosed with WT between 1st January 2017 to 31^st^ January 2021.

### Study setting

The study was conducted in the surgical and pediatric departments, pediatric surgery, and pediatric oncology units of MRRH.

### Inclusion criteria

Children with a histologically confirmed diagnosis of WT.

### Exclusion criteria

Children with missing data on clinical and pathological characteristics and children involved in the pilot study.

### Study procedure

Permission to access the records was sought from the research ethics committee. The data extraction tool was designed following the variables to be studied. Electronic in-patient numbers of children diagnosed with WT between January 2017 to January 2021 at MRRH were accessed from the open medical record system (MRS). These electronically retrieved in-patient numbers were used to trace paper charts from the records office of the pediatric surgery and oncology unit. Unique patient codes were assigned to each patient’s file, to maintain patient confidentiality. The data extraction tool was then used to capture clinical, imaging, histology, and treatment records from the paper charts. Any missing information from the paper charts was obtained by cross-checking with the electronic database. A sub-database was created from the data extracted for analysis.

### Analysis

A complete dataset was exported into STATA software version 15.0 for analysis. Continuous data were summarized into means, standard deviations, median, and interquartile ranges. Proportions for ordered categorical data were done and results were presented as percentages. Clinical and histology characteristics were described as frequencies and percentages. One-year overall survival was estimated using the Kaplan–Meier curve and expressed as a percentage with its corresponding 95% confidence interval. To identify predictors of OS, data was declared as survival time data, and time of survival was used as the time variable. In bivariate analysis, cox proportional hazard model regression was used to establish the covariates of time of survival. The unadjusted hazard ratios with their corresponding 95% Confidence intervals were reported for each covariate. A significance level of 5% was considered.

## Results

### Clinical characteristics

**Fig. 1:**
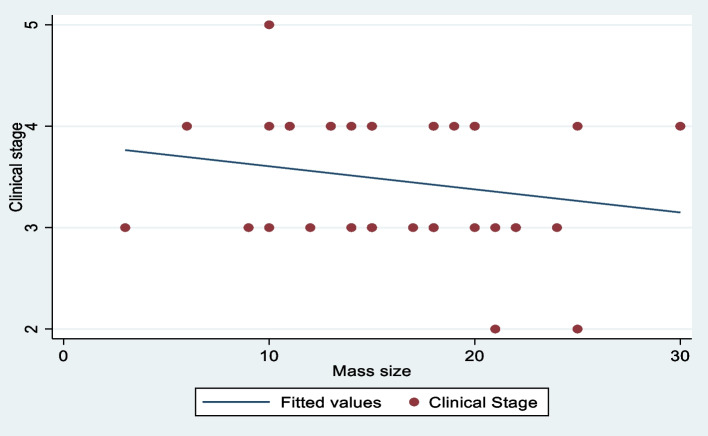
Correlation of tumor size by clinical stage

Out of 41 records of children studied, 27(65.9%) were below 5 years of age, and 14(34.1%) were above 5 years of age. Females being the majority diagnosed with WT, were 23(56.1%). All 41 (100%) children presented with symptoms of abdominal swelling and pain. Sixteen (39%) children had severe weight loss, 15 (36.6%) had hypertension and 19 (46.3%) children had developed temperature on admission. In our study, the average duration of presentation with WT was 2 months. The cardinal presenting symptoms of WT were; abdominal swelling, hypertension, and hematuria observed in only 3(7.3%) children. No associated predisposition syndromes were noted. Nineteen children were diagnosed with stage III (46%) and IV (46%) of WT. Only 2 (5%) children presented with stage II WT and one (3%) with stage V. There was no child diagnosed with stage I WT at admission (Table 1). There was, however, no correlation between the WT stage and the size of the tumor (Fig. 1).

**Table 1:** Clinical characteristics of children diagnosed with Wilms Tumor

Parameters		Frequency	Percentage (%)
Demographics			
Age	< 5 years	27	**65.9**
	> 5 years	14	34.1
Sex	Male	18	**43.9**
	Female	23	**56.1**
Clinical features	Abdominal swelling	41	**100**
	Abdominal pain	41	**100**
	Difficulty breathing	4	9.7
	Drenching sweat	2	4.8
	Hematuria	3	7.3
	Wasted (< -3SD)	16	**39**
	Hypertension	15	**36.6**
	Raised temperature	19	**46.3**
Sites of metastasis	Liver	5	12.2
	Lungs	7	17
	Spleen	1	2.4
	Liver + lungs	4	9.7
Stages of Wilms Tumor	II	2	5
	III	19	**46**
	IV	19	**46**
	V	1	3
Side of tumor origin	Right	27	**65.9**
	Left	14	**34.1**
Treatment/Investigations			
Chemotherapy	Received	36	**87.8**
	Did not receive	5	12.2
Surgery(nephrectomy)	Performed	36	**87.8**
	Not performed	5	12.2
Resection margin status	Positive	13	31.7
	Negative	14	34.1
	Not assessed	14	34.1
Radiotherapy	Received (from Mulago Hospital)	5	12.1
	Did not receive	36	**87.8**
Compliance to chemotherapy	Received all cycles, on schedule	20	48.8
	Received all cycles, not on the schedule	8	19.5
	Missed some cycles	13	31.7
	Loss to follow up	15	36.6
Outcome of treatment	Remission	9	22
	Partial response	9	22
	Progressive WT	15	36.5
	Relapse	8	19.5

Sites of metastasis: Lung metastasis accounted for 7 (17%) cases, liver 5 (12.2%), and spleen 1 (2.4%) cases. Four (9.7%) of children had metastasis to both the liver and lungs.

Side of tumor origin: WT originated from the right kidney in 27 (65.8%) of children, and the left in 14 (34.1%) of children diagnosed with WT.

Of the children diagnosed with WT, 41 (100%) had chemotherapy, 36 (87.8%) had surgery, and 5 (12.2%) did not get surgery. Of the 36 children who received chemotherapy, 20 (48.8%) complied and received all cycles on schedule, 8 (19.5%) received chemotherapy but not on schedule, and 13 (31.7%) missed some cycles of chemotherapy. Those who had nephrectomy had 13 (31.7%) with residual tumor cells and 14 (34.1%) without residual tumor cells in the perinephric fat. Samples that did not have their resection margins assessed were 14 (34.1%). There was however an imbalance in the multimodal treatment, where 36 (87.8%) of children who received surgery and chemotherapy did not receive radiotherapy, as only 5 (12.2%) received them from Mulago national referral hospital (MNRH). Following chemotherapy and surgery alone, 8(19.5%) relapsed (Table 1).

### Histological characteristics of Wilms Tumor

Thirty-one (75.6%) of children diagnosed with WT had favorable histology, while 10 (24%) had unfavorable histology. Out of the 10 children who had anaplasia, 7 (70%) had diffuse and 3 (30%) had focal anaplasia. Five (12.1%) children had blastemal predominant, 26 (63.4%) had triphasic, and 10 (24.4%) had stromal predominant subtypes of WT**.** There was no predominant epithelial WT subtype (Table 2).

**Table 2:** Histological characteristics of Wilms tumor

Histology description	Frequency	Percentage (%)
**Favorable (No anaplasia)**		
Blastemal predominant	5	12.1
Stromal predominant	10	24.5
Triphasic	26	63.4
**Unfavorable (Anaplasia)**		
No anaplasia	31	75.6
Diffuse anaplasia	7	17.1
Focal anaplasia	3	7.3

### One-year overall survival

**Fig. 2:**
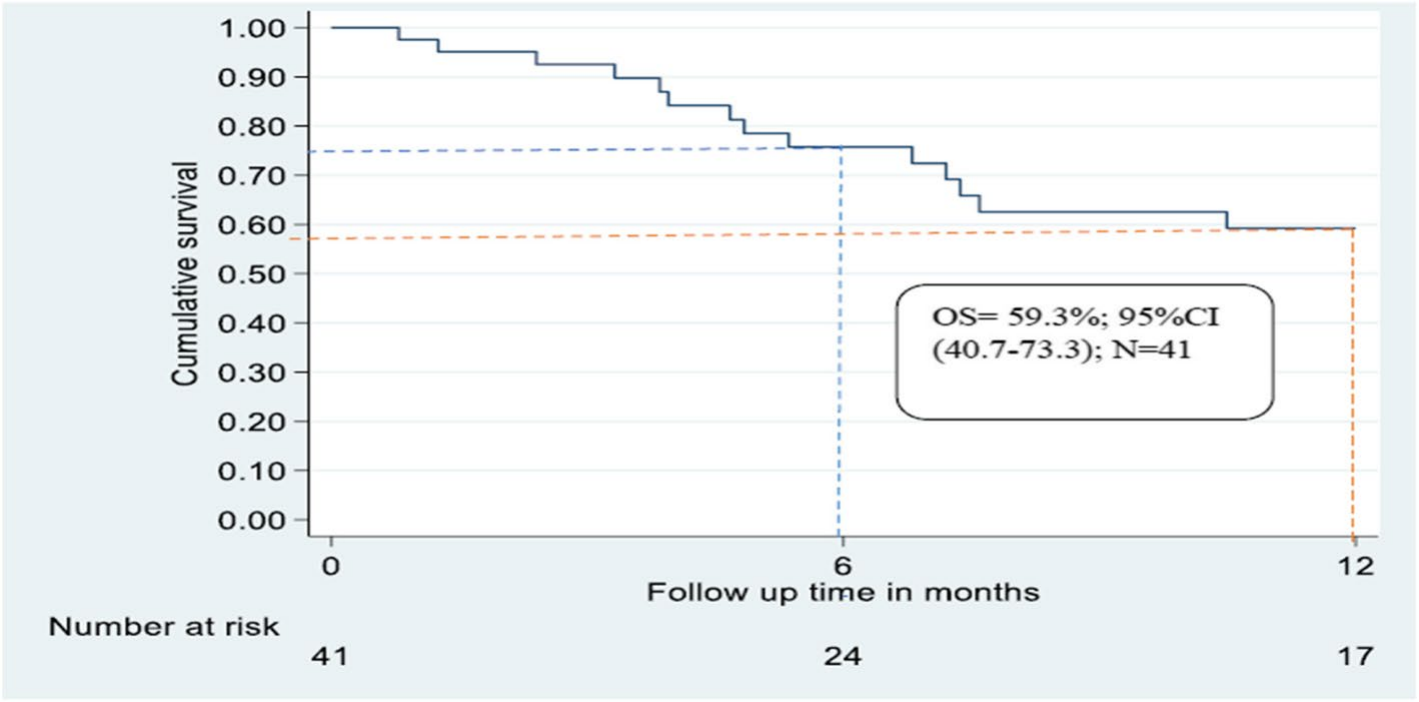
Kaplan–Meier estimate of overall survival

At the beginning of the follow-up, one child died within the first month, making the survival probability initially 97.6% (95% CI: 83.9–99.7). The survival rate decreased subsequently over the next eleven months, being 75.7% (95% CI: 0.5843- 0.86606) at 6 months, with 9 deaths and 8 losses to follow up (Fig. 2). At the end of one year, the overall survival was 59.3% (95% CI: 40.7–73.3) and a total of 14 children had died, 17 were alive, and 10 got lost to follow-up.

### Overall survival in children with favorable Versus unfavorable tumor

**Fig. 3:**
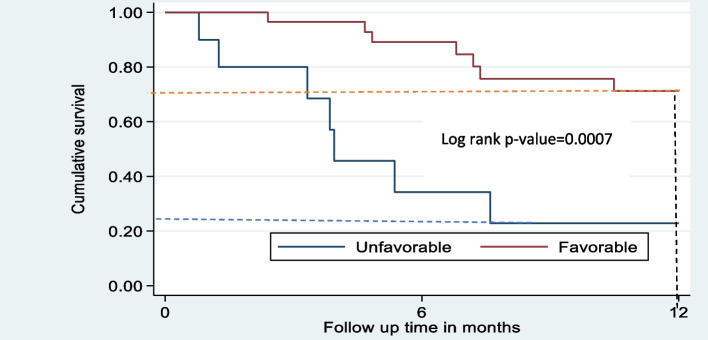
Cumulative overall survival by favorable versus unfavorable histology

The one-year OS in children with the favorable histology WT type was 72%, which was threefold the survival rate of those with the unfavorable histology WT type at 22% (Fig. 3)**.** However, by the end of one year, 15 children with favorable and only 2 unfavorable WT histology were still at risk of death.

### One-year OS according to WT size

**Fig. 4:**
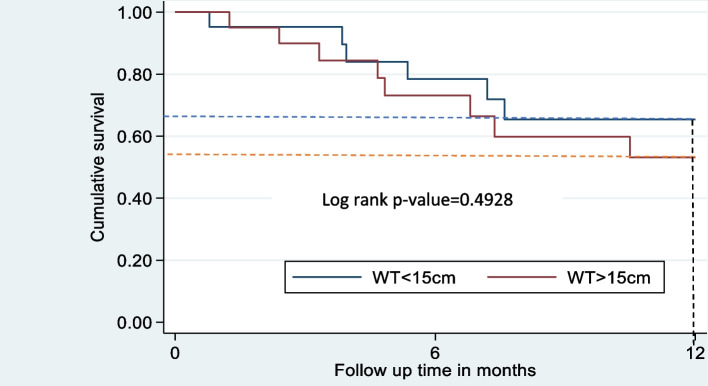
Cumulative survival by Wilms Tumor size

Children with WT size < 15 cm had OS of 68%, while WT > 15 cm decreased OS to 58% (Fig. 4). Nine children having a WT size of < 15 cm and eight with a WT size of > 15 cm were at risk at the end of one year.

### Overall survival according to the WT stage

**Fig. 5:**
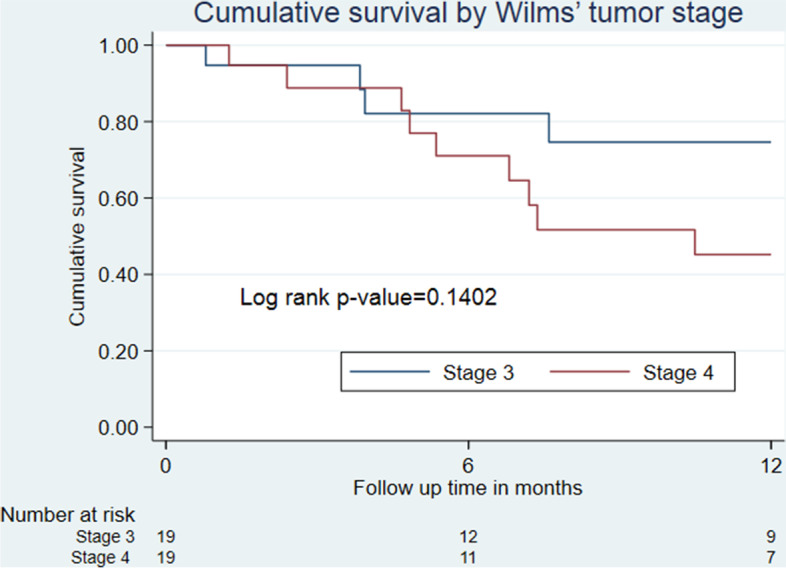
Cumulative Overall survival by Wilms tumor stage

Children diagnosed with stage 3 WT had OS at 78%, only second to those diagnosed with stage 5 WT at 100%. However, only 1 child had WT stage 5 and remained at risk throughout the follow-up period. OS in WT stage 4 was the lowest at 48%. The majority, 92.6% of newly diagnosed children had stage 3 and 4 WT (Fig. 5). Only 2 children were diagnosed with WT 2, and none with stage 1, an insignificant number that could not be correlated to represent OS by tumor stage.

### Predictors of one-year overall survival

Variables were analyzed in the bivariate model to rule out confounders (Table 3). Unfavorable histology type was found to increase the risk of death from WT by 5.1 times (95%CI: 1.77–92.50), while tumor size above 15 cm was found to increase the risk of death from WT by 6 times (1.32–34.95). Other variables were found not to significantly affect survival after adjusting for confounding at all levels of analysis.

**Table 3:** Bivariate and multivariate analysis for predictors of overall survival

Predictor variables	Categories	CrudeHazard Ratio (95% C.I)	*p*-value	AdjustedHazard Ratio (95% C.I)	*p*-value
**Age**	< 5 years	1		1	
	≥ 5 years	1.50 (0.52–4.34)	0.452	0.539 (0.10–2.78)	0.461
**Sex**	Male	1		1	
	Female	1.59 (0.53–4.77)	0.404	1.234 (0.26–5.71)	0.788
**Wilms tumor stage**	Stage 3	1		1	
	Stage 4	2.366 (0.72–7.70)	0.152	3.312 (0.77–14.21)	0.107
**Histology type**	Favorable	1		1	
	Unfavorable	5.16 (1.79–14.89)	**0.002**	73.836(8.13–669.83)	0.000^*^
**Treatment response**	No relapse	1		1	
	Relapse	1.46 (0.50–4.22)	0.481	1.164 (0.25–5.34)	0.844
**Surgery**	Nephrectomy done	1		1	
	Nephrectomy not done	1.763 (0.22–13.55)	0.586	0.152 (0.005–4.01)	0.260
**Compliance to chemotherapy**	Received on schedule	1		1	
	Not received on schedule	0.646 (0.21–193)	0.435	0.351 (0.06–1.98)	0.236
**Tumor size**	≤ 15 cm	1		1	
	> 15 cm	1.44 (0.50–4.16)	0.495	5.87 (1.04–33.03)	0.044^*^

^*^Significant at p < .05

### Treatment approach to Wilms tumor

**Fig. 6:**
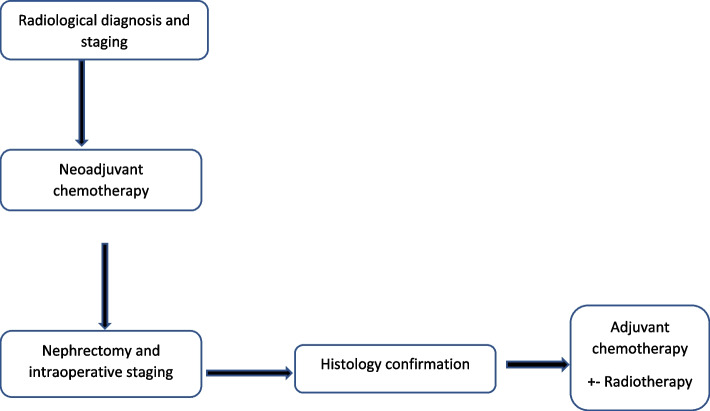
SIOP treatment approach of WT at MRRH

Preoperatively, all children radiologically diagnosed with either favorable or unfavorable WT type receive chemotherapy according to the SIOP protocol, depending on the WT stage, as described below. The flow of diagram of the treatment approach used at MRRH is shown on Fig. 6.

For FH stages 1 and 2 are treated with vincristine and dactinomycin (VD), or Actinomycin and doxorubicin (AD) combinations. Vincristine and dactinomycin are administered for WT stages above 3.

WT stages 2–4 with UH are treated with cyclophosphamide, ifosfamide, carboplatin, and etoposide, in addition to radiotherapy. However, stage 5 UH is treated with vincristine, dactinomycin and doxorubicin for 6–12 weeks, after which nephron-sparing surgery is performed [16, 17].

Post nephrectomy, all children receive adjuvant chemotherapy except for those with FH stage 1.

Children with stages 2 and above with FH receive vincristine, actinomycin, and doxorubicin.

In addition to radiotherapy, those with UH are treated with cyclophosphamide, doxorubicin, etoposide, and carboplatin for 27 weeks, with each cycle of chemotherapy being administered at a 21-day interval [16, 18].

## Discussions

### Clinical characteristics of children diagnosed with Wilms Tumor

In this study, the majority (65.9%) of children diagnosed with WT were below 5 years of age. The age most affected correlates with other studies [19, 20]. Children below 5 years of age were affected most probably because of genetic or embryological predispositions.

There were more female children diagnosed with WT in this study than males, with a Male: Female ratio of 1:1.2, which correlates with a study done in Rwanda by [21], which showed more females diagnosed with WT than males. We attribute this female predominance to WTX gene present on the dominant X-chromosome, which is easily inactivated by a single point mutation. This single hit monoallelic inactivation makes it more susceptible to mutation. Because X-chromosome is not dominant in males, this could explain the fewer number of males affected [22]. Gender predominance of children diagnosed with WT has shown variations in many kinds of literature. Studies done in the United Kingdom [23], and in Nigeria [24] all show the contrary, with male predominance over females.

In this study, the most common symptoms at presentation in all children were abdominal pain and swelling. All children presented with abdominal swelling probably because any small increase in the tumor size would have decreased the already small intrabdominal cavity volume in children. The pressure effect from this swelling would subsequently cause abdominal pain. The above symptoms were also found in studies done in America [25], and Nigeria [26].

Hypertension was one of the common presenting symptoms in children with WT, due to hypersecretion of renin by the tumor [27] though the number was lower than in a study done in America, where 63% of children presenting with WT had hypertension [28]. The disparity in hypertension incidence could be explained by high rates of overweight and obesity [29]. In this study, most children (95%) presented with stage 3 and above, of WT, probably due to the rapid progression of WT, despite the mean presentation duration of 2 months. A similar finding has been found in Nigeria [9], as well as Rwanda [21]. Late presentation could have been caused by a delay at home because the tumor exhibits a painless pattern yet progressively grows.

Many children (87.8%) did not receive chemotherapy, probably because they could not foot the costs of transport to Mulago national referral hospital (MNRH), the only hospital in the country with a radiotherapy machine.

### Histological characteristics of Wilms Tumor

The majority (75.6%) of children diagnosed with WT had a favorable (non-anaplastic) histological type, similar to a finding in Nigeria [24]. Comparatively, only 24% had an unfavorable subtype of WT, a percentage which was more compared to a study done in Iran, where children diagnosed with WT had 10% of the anaplastic subtype [11]. The higher percentage of anaplasia in our setting could be due to treatment default, as well as limited access to radiotherapy. Defaulting treatment causes intracellular molecular changes which transform a cell from a favorable to anaplastic form. In this study, the majority of WT diagnosed on admission (68.8%) had their origin from the Right kidney and all (100%) were unilateral, in keeping with published data [25]. The occurrence of WT mostly in the right kidney contradicts research done in Nigeria, where 63.3% of WT affected mostly the left kidney [24]. The difference could be attributed to the 5 years duration of study in the Nigerian study. 8 (19.5%) who did not receive chemotherapy on the schedule were probably due to a delay in first treating other associated nosocomial diseases/illnesses like severe malaria or malnutrition before the resumption of chemotherapy. Those who missed some cycles of chemotherapy could have been hindered by long distances to the hospital, coupled with financial constraints.

### One-year overall survival

This was found to be 59.3%, which is a better survival rate compared to those reported in MNRH. We attribute the better 1-year OS to the fact that the majority of children had favorable histology. The better 1-year OS could also be because all children in the study had unilateral WT, which has been shown not to affect OS greatly even in stage IV [23]. In our setting prompt multidisciplinary management of these children by all departments could as well as be the reason for a fair OS found in this study. Despite better OS found in this study, overall survival is still low in our setting compared to high-income countries where OS surpasses 80% [6]. Lower OS in our setting could be attributed to challenges leading to late-stage presentation and interrupted treatment of children especially delay in timely surgical intervention. Although many factors would be attributed as barriers to overall survival, we think that lack of funds plays a great part in limiting access to care and loss of follow-up. Due to a lack of funds for transport, children miss their chemotherapy schedules which predispose to tumor resistance and recurrence, which impacts negatively on survival. Lack of funds also limits early investigations which delay the initiation of treatment. Since the funds are needed to facilitate the child’s stay in the hospital in terms of feeding and doing investigations like CT scans, and compounded by frustrations of delayed surgery due to shortage of pediatric surgeons, limited resources, and the myth that cancer is incurable, they opt not to return to continue with treatment.

Children with unfavorable WT histology had poor OS compared to those with favorable type, similar to previous studies done in Iran [11] and in most parts of Africa [7, 24]. The poor prognosis is due to the anaplastic cellular morphology of unfavorable WT type which increases resistance to chemotherapy [30], predisposing to recurrence and tumor metastasis.

Wilms tumor size > 15 cm decreased OS to 58%, probably because of metastasis and tumor rupture, which are risks in huge tumors. In literature, WT size > 10 cm greatly decreases OS [10].

Similar to findings in Nigeria [9] and Kenya [31], the advanced stage of WT was found in our study to cause poor OS. The late-stage disease is associated with complications like hypertension, anemia due to hematuria, urinary tract infections, and metastasis [32], all of which predispose to multiorgan failure to greatly decrease OS. The discrepancy of stage 2 having poor OS second to stage 4 could be attributed to only 2 children being diagnosed with WT at this stage. WT stage 4 had excellent OS at 100% probably because only 1 participant was diagnosed at this stage. However, this OS can be expected even with advanced WT stages, similar to findings in other studies [7, 13].

### Predictors of survival

Tumor size greater than 15 cm and unfavorable histology were two factors that predicted overall survival. Tumor size greater than 15 cm was found to contribute to poor OS, probably due to tumor destruction to the affected kidney, causing renal failure. A study in Germany [10], showed tumor sizes more than 10 cm to be associated with poor survival. This slight difference could be because many children in our study had late-stage WT at presentation, compared to those in the study conducted in Germany, where the majority had stage one WT. Unfavorable histology in our study was shown to reduce overall survival. This is probably because this type of histology poorly responds to chemotherapy. Similar findings were found in studies done in America [33] and New Mexico [34].

## Conclusions

Overall survival of WT at MRRH was found to be 59.3%, and predictive significant factors noted were unfavorable histology and tumor size greater than 115 cm. Unfavorable WT histology and late stages of WT correlated with poor OS.

## Data Availability

The datasets used and/or analyzed during the current study available from the corresponding author on reasonable request.
